# Endoscopic full-thickness resection of a large gastric schwannoma and iatrogenic cervical esophageal perforations: A case report

**DOI:** 10.1097/MD.0000000000038808

**Published:** 2024-07-12

**Authors:** Siying Huang, Sifu Huang, Taiyong Fang

**Affiliations:** a Department of Gastroenterology, The Second Affiliated Hospital of Fujian Medical University, Quanzhou, Fujian, P.R. China

**Keywords:** cervical esophageal perforations, endoscopic full-thickness resection, gastric schwannomas

## Abstract

**Introduction:**

Gastrointestinal schwannomas are most commonly found in the stomach. Owing to their nonspecific clinical and endoscopic presentations, distinguishing gastric schwannomas (GS) from other gastric submucosal tumors based on typical symptoms and endoscopic features is challenging. Endoscopic full-thickness resection (EFTR) is safe and effective for GS management; however, no standard method exists for the extraction of large gastric specimens after endoscopic treatment.

**Case presentation:**

We report the case of a 72-year-old Chinese woman who presented with abdominal distension.

**Diagnosis, interventions, and outcomes:**

Gastroscopy revealed a submucosal bulge on the anterior wall of the lower stomach near the greater curvature. Endoscopic ultrasonography and computed tomography suggested a stromal tumor. The patient underwent EFTR of the stomach, and the tumor was successfully removed. The surgical specimen, with a long-axis diameter of approximately 5.5 cm in vitro, was extracted using a snare. Subsequent endoscopic examination revealed longitudinal, full-thickness perforations > 2 cm at the esophageal entrance. Over 10 metal clips were used to seal the mucosa, and a gastrointestinal decompression tube was placed. Follow-up radiography performed at 1 week postoperatively revealed an esophageal mediastinal fistula, which required subsequent endoscopic intervention to close the fistula using metal clips. The patient showed improvement and was discharged at 3 weeks postoperatively. Follow-up esophageal radiography revealed no abnormalities. Postoperative immunohistochemical analysis indicated CD34 (−), CD117 (−), DOG-1 (−), Ki67 (1%), S-100 (+), SDHB (+), SOX-10 (+), and Desmin (−), confirming the diagnosis of GS. Three months postoperatively, gastroscopy showed that the esophageal perforation healed well, a white ulcer scar had formed locally, metal clips were found in the stomach body, and no recurrence was found.

**Conclusion:**

EFTR is effective for removing giant schwannomas, although the extraction of large specimens may result in iatrogenic cervical esophageal perforations. Perforations > 2 cm can be managed using endoscopic metal clip closure.

## 1. Introduction

Gastrointestinal schwannomas are relatively rare. Gastric schwannomas (GS) are the most prevalent type of gastrointestinal schwannomas and are typically located in the gastric body.^[1,2]^ Distinguishing between GS and gastric stromal tumors (GST), which are both submucosal tumors (SMTs), is often challenging using preoperative ultrasonography and computed tomography (CT).^[3]^ The gold standard for diagnosing schwannomas is an immunohistochemical examination of S-100, with approximately 97.9% of schwannomas testing positive for S-100.^[4]^

Endoscopic full-thickness resection (EFTR) is safe and effective for GS management.^[5,6]^ However, no standard method exists for the extraction of large gastric specimens after endoscopic treatment. Esophageal perforations are mostly attributed to iatrogenic causes, occurring in the cervical esophagus in approximately 15.2% of cases.^[7]^ The mortality rate for cervical esophageal perforations (CEPs) is 6% to 8%.^[8]^ Perforations ≤ 2 cm may be repaired via through-the-scope or over-the-scope endoscopic clips, while perforations > 2 cm may require stent placement or surgical suturing (primary repair with a muscle flap).^[9]^

This case report describes the use of EFTR for the complete removal of a giant submucosal mass in the gastric body, which was later identified as GS using immunohistochemistry. However, CEPs (>2 cm) occurred during specimen extraction. After 2 endoscopic closures with metal clips, the patient ultimately recovered and was discharged. This report highlights the use of EFTR for the complete excision of a giant GS, despite the risk of CEPs during postoperative removal of large specimens. Endoscopic closure is a viable option for CEPs > 2 cm, although further research is needed to establish its efficacy in the management of CEPs.

## 2. Case presentation

**Figure 1.**
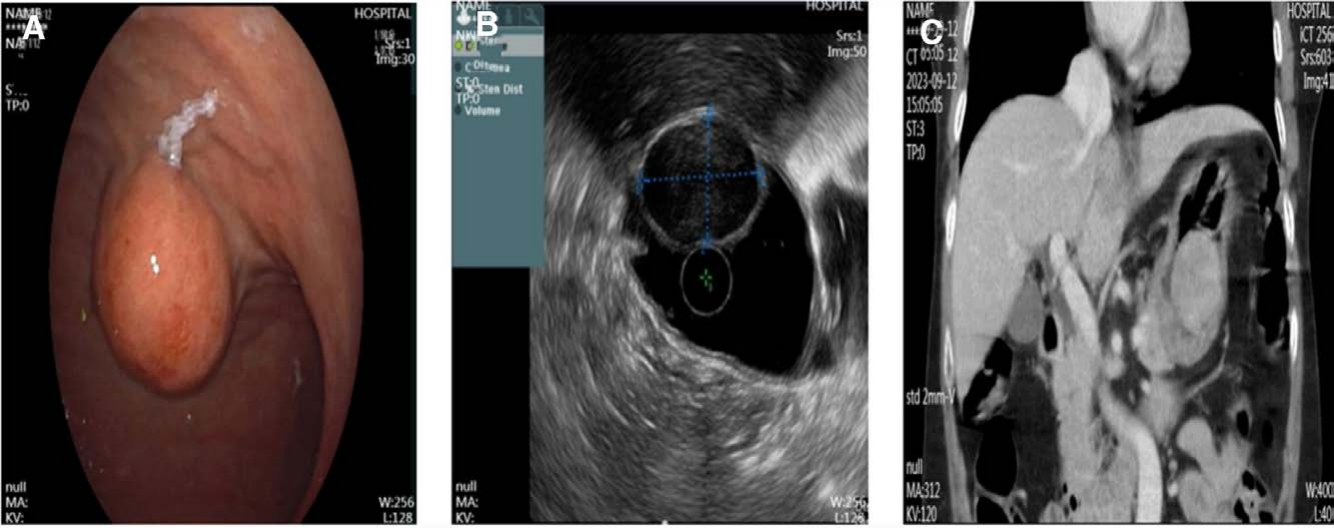
Abdominal computed tomography and endoscopic ultrasonography. (A) White-light endoscopy shows a submucosal bulge on the anterior wall of the lower stomach near the greater curvature. (B) The lesion originates from the muscularis propria and appears as an uneven hypoechoic mass that is primarily intraluminal with no detectable blood flow signals on ultrasonography. (C) Computed tomography scan reveals a quasi-circular mass of slightly lower density on the anterior wall of the stomach with clear boundaries, uniform density, and uniform enhancement with contrast.

**Figure 2.**
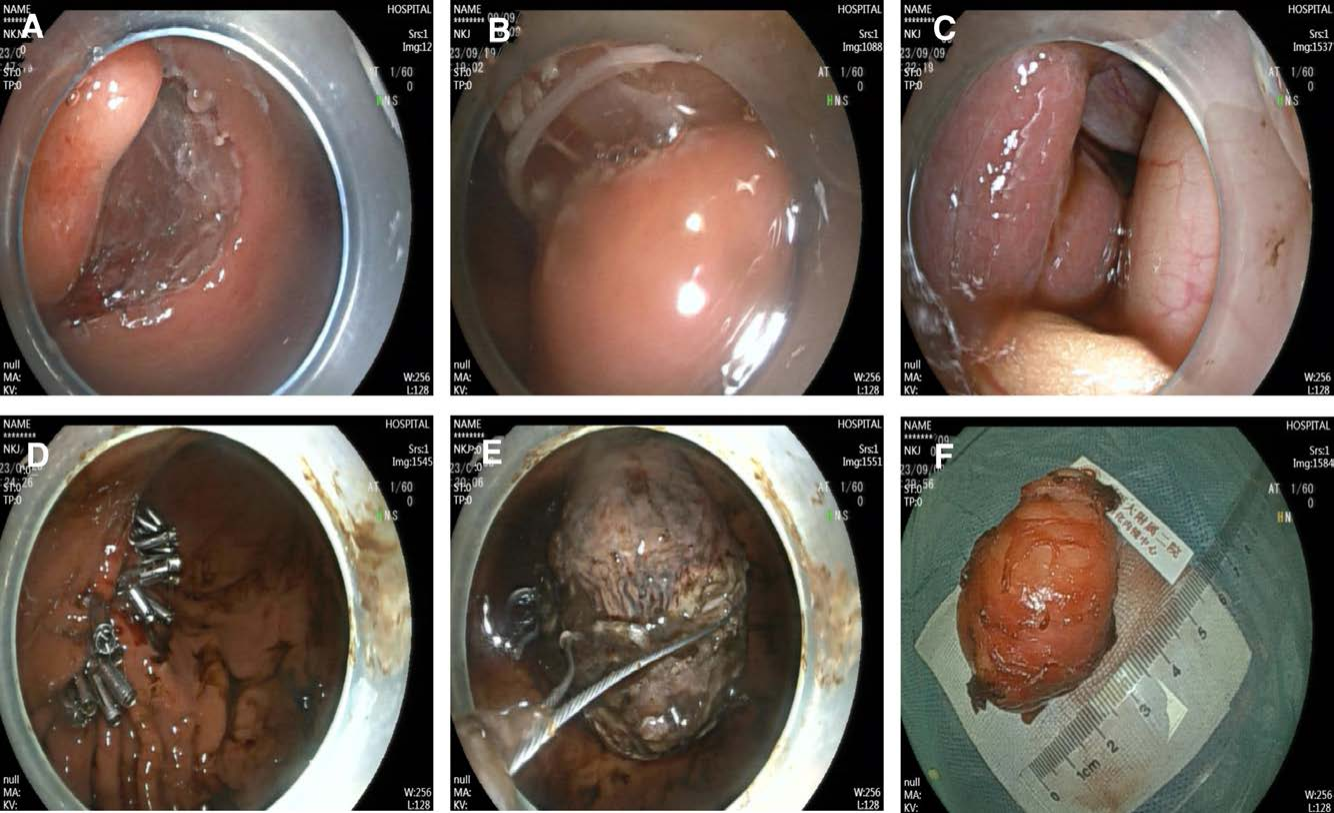
Endoscopic full-thickness resection. (A) The lesion is peeled off along the base of the tumor. (B) A snare is inserted into the lesion to draw out the tumor body and expose the visual field. (C) The lesion is completely resected, and there is a muscular defect (2.5 cm in diameter) at the base of the wound. (D) More than 20 metal clips are placed to seal the wound surface completely. (E) A snare is used to remove the tumor. (F) The length of the tumor is measured at about 5.5 cm in vitro.

**Figure 3.**
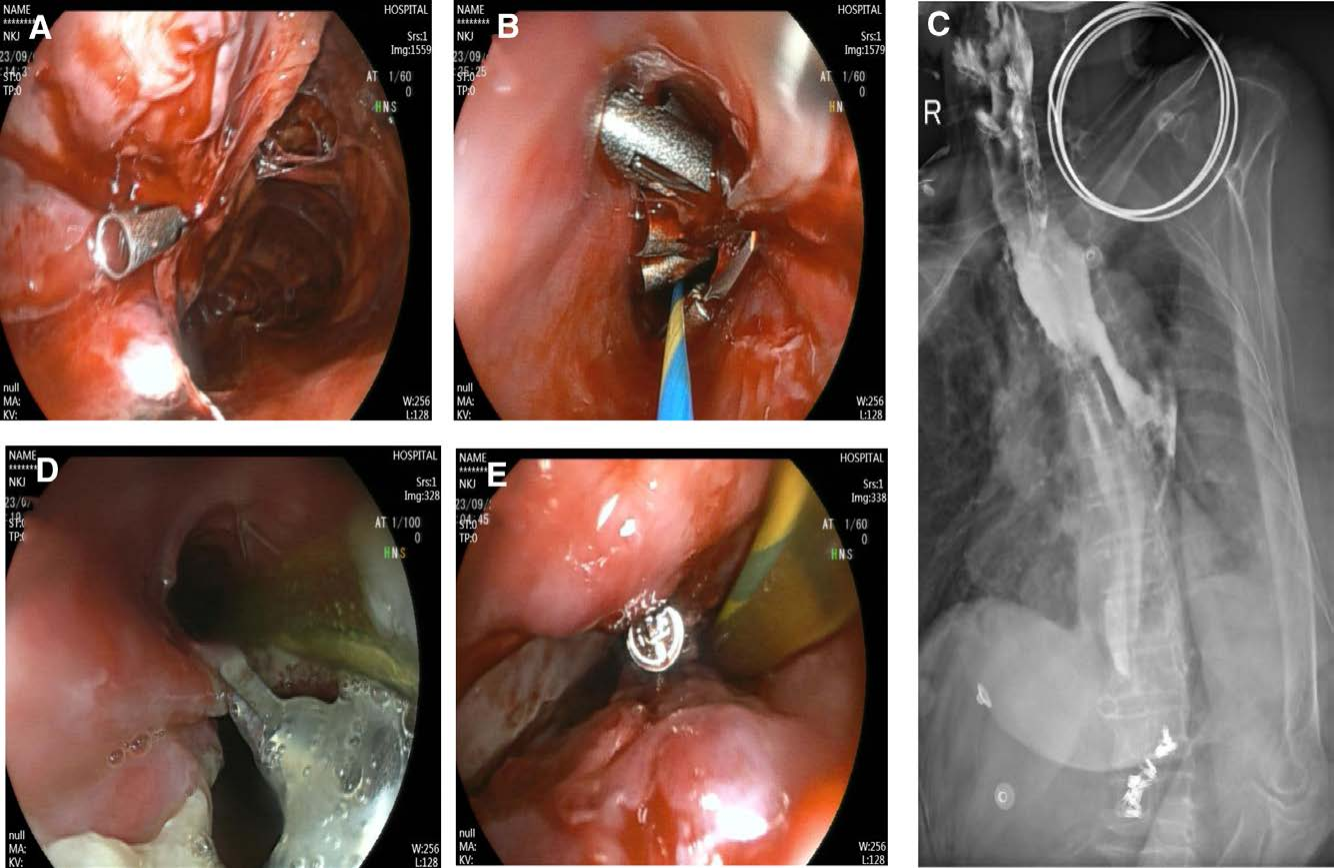
Endoscopic closure of the fistula. (A) The cervical esophageal perforations due to specimen extraction after endoscopic full-thickness resection. (B) The first endoscopic closure of the esophageal fistula with metal clips. (C) The first esophagography reveals an esophageal mediastinal fistula. (D) The second gastroscopy reveals partial healing of the fistula. (E. The second endoscopic closure of the esophageal fistula with metal clips.

**Figure 4.**
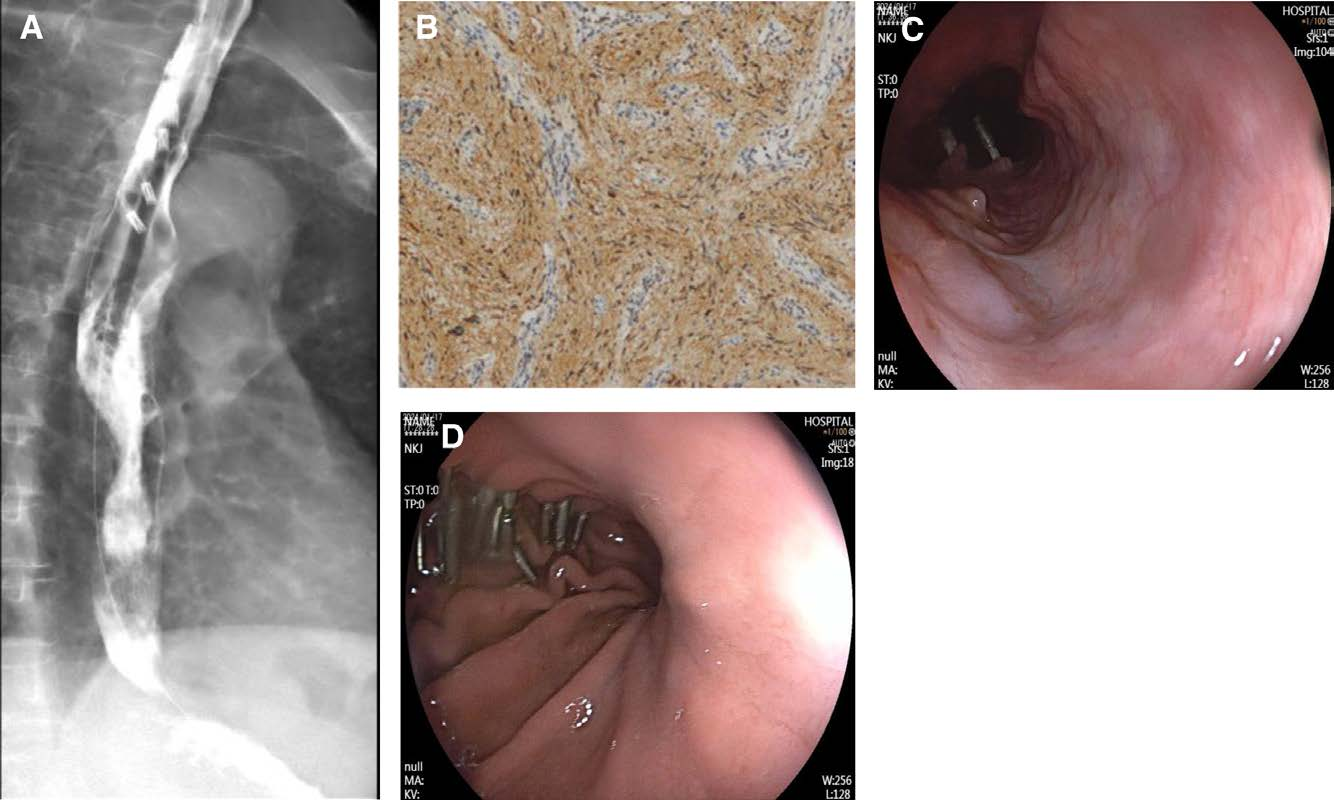
Treatment outcomes and follow-up. (A) The second esophagography reveals no esophageal mediastinal fistula. (B) Immunohistochemistry examination is positive for S-100 (100× magnification). (C) Gastroscopy showing that the esophageal perforation healed well and a white ulcer scar has formed locally. (D) Metal clips are found in the stomach body, but no recurrence is found.

A 72-year-old Chinese woman who presented with abdominal distension underwent gastroscopy, which revealed a submucosal bulge on the anterior wall of her lower stomach near the greater curvature (Fig. 1A). Endoscopic ultrasonography revealed that the lesion originated from the muscularis propria and appeared as an uneven, hypoechoic mass with a regular shape. The mass was completely encapsulated and primarily intraluminal, measuring 2.6 × 3.4 cm at its largest section, with no detectable blood flow signals (Fig. 1B). A CT scan revealed a lesion measuring approximately 3.81 × 3.29 cm with slightly lower density, clear boundaries, and uniform enhancement with contrast (Fig. 1C). Both CT and endoscopic ultrasonography suggested GST diagnosis. The patient underwent EFTR of the stomach. The operative time was about 3 h. At first, the lesion was marked using electrocoagulation with a one-off mucosal incision at the base of the lesion. A mixture of normal saline and methylene blue was injected submucosally at the base of the lesion, and a one-off mucosal incision was performed to circumferentially cut the mucosal layer along the marked points to expose the tumor (Fig. 2A). The lesion was peeled off along the base of the tumor, and electrocoagulation was performed to expose the blood vessels and bleeding points during the operation. The relationship between the lesion and deep muscle layer was close. A snare was inserted into the lesion to draw out the tumor body and expose the visual field (Fig. 2B), and the lesion was completely resected. A muscular defect (2.5 cm in diameter) was found at the base of the wound (Fig. 2C) and more than 20 metal clips were placed to completely seal the wound surface (Fig. 2D). After resection, a snare was used to remove the tumor (Fig. 2E). The length of the tumor was about 5.5 cm in vitro (Fig. 2F). A subsequent endoscopic examination revealed longitudinal full-thickness perforations at the esophageal entrance. More than 10 metal clips were used to fix the mucosal damage, and a gastrointestinal decompression tube was placed (Fig. 3A and B). Follow-up radiography performed 1 week postoperatively revealed an esophageal mediastinal fistula (Fig. 3C), which was treated via subsequent endoscopic intervention with metal clips (Fig. 3D and E). After fasting, anti-infection therapy, and enteral nutrition, an esophagography conducted 3 weeks postoperatively showed no abnormalities (Fig. 4A). Ultimately, after 21 days in hospital, the patient recovered and was discharged. Postoperative immunohistochemical analysis revealed CD34 (−), CD.117 (−), DOG-1 (−), Ki67 (1%), S-100 (+), SDHB (+), SOX-10 (+), and Desmin (−), confirming GS diagnosis (Fig. 4B). Three months after the operation, the patient recovered well, and gastroscopy showed that the esophageal perforation healed well, a white ulcer scar had formed locally, metal clips were found in the stomach body, and no recurrence was found (Fig. 4C and D).

## 3. Discussion

Patients with GS typically do not have obvious symptoms, and conditions are often discovered incidentally after complications, such as gastrointestinal bleeding or abdominal discomfort. GS, generally considered a benign tumor with low potential for malignant transformation, is distinguished from GST using immunohistochemistry for S-100.

In 2011, Zhou et al^[5]^ reported the use of EFTR to treat GST, using an active perforation technique to achieve full-layer tumor resection. Since then, EFTR has become widely used during endoscopic treatments, as several clinical studies have established the safety and efficacy of EFTR for GST.^[10,11]^ Before using EFTR, GSTs < 3 cm that were primarily intraluminal were treated endoscopically. However, endoscopic treatment has been reported as safe and effective for GSTs with a maximum diameter of 3 to 5 cm. Xiang et al^[12]^ demonstrated the safety and efficacy of endoscopic treatment for GSTs with a diameter ≥ 3 cm and volume < 125 cm^3^. Digestive tract SMTs with a maximum diameter > 5 cm have also been treated via EFTR.^[13]^ Additionally, EFTR has been used to treat GS,^[5]^ with a 5-year study from a major tertiary center in China confirming that EFTR is safe and effective for GS management (mean tumor size, 22.9 ± 15.1 mm).^[6]^

Complete tumor resection minimizes the risk of residual tumors and recurrence.^[14]^ Adherence to the tumor-free principle during endoscopic resection maintains capsular integrity and is essential for successful treatment. Therefore, complete specimen extraction after the endoscopic treatment of giant gastric tumors is essential. According to the American Society for Gastrointestinal Endoscopy guidelines, lesions > 4 cm remain challenging when using any endoscopic approach.^[15]^ However, no standard method for extracting large gastric specimens after endoscopic treatment has been established. The method of foreign body extraction in the upper digestive tract should be used for reference. For example, retrieval graspers can be useful for retrieving soft objects, endoscopic baskets may be useful for round objects, and retrieval nets or bags can provide a more secure grasp. Using a protective device, such as overtubes, a transparent cap, or latex rubber hood, could prevent esophagogastric/pharyngeal damage during endoscopic extraction of large specimens.^[16]^ In a case report, the balloon-assisted technique was used to extract giant GST specimens after endoscopic resection.^[17]^ If the specimen cannot be removed using endoscopy, a combination of endoscopic and laparoscopic techniques should be considered.^[18]^

This report highlights that extracting a specimen from a giant gastric tumor may lead to CEP. Esophageal perforation is a rare yet potentially life-threatening condition with a relatively high mortality rate.^[19]^ Particularly, the mortality rate of CEP is 6% to 8%.^[8]^ The most common cause of esophageal perforation is iatrogenic, with approximately 15.2% of perforations occurring in the cervical esophagus.^[7]^ The management of iatrogenic CEPs remains challenging. Perforations ≤ 2 cm may be repaired using through-the-scope or over-the-scope endoscopic clips, while perforations > 2 cm may require stent placement or surgical suturing (primary repair with a muscle flap).^[9]^ Proximal stent placement may cause severe discomfort due to proximity to the upper esophageal sphincter and is traditionally considered a relative contraindication for upper esophageal perforations. As the perforation was > 2 cm in this case, endoscopic titanium clip closure was performed, and the patient’s outcome was favorable, suggesting that endoscopic closure is a viable option for treating CEPs > 2 cm.

In conclusion, EFTR is technically feasible for the complete resection of giant GS. However, the postoperative extraction of large specimens must be thoroughly considered preoperatively. Due to the lack of standardized methods for specimen extraction, the method should be individualized to the patient. Endoscopic closure is a viable option for CEPs > 2 cm due to specimen extraction, although further research is required to evaluate its efficacy for the treatment of CEPs. To minimize the occurrence of similar complications, we suggest that EFTR for giant GS should be performed by appropriately trained advanced therapeutic endoscopists with rich experience in large gastrointestinal foreign body extraction.

## Acknowledgments

We would like to thank Editage (www.editage.cn) for English language editing.

## Author contributions

**Resources**: Siying Huang.

**Writing – original draft**: Siying Huang, Sifu Huang, Taiyong Fang.

**Writing – review & editing**: Siying Huang, Sifu Huang, Taiyong Fang.
